# Environmental impact assessment based on particulate matter, and chlorophyll content of urban trees

**DOI:** 10.1038/s41598-024-70664-4

**Published:** 2024-08-28

**Authors:** Vanda Éva Abriha-Molnár, Szilárd Szabó, Tibor Magura, Béla Tóthmérész, Dávid Abriha, Bianka Sipos, Edina Simon

**Affiliations:** 1 HUN-REN–UD Anthropocene Ecology Research Group, University of Debrecen, Egyetem sq. 1, Debrecen, 4032 Hungary; 2 Department of Ecology, Faculty of Science and Technology, University of Debrecen, Egyetem sq. 1, Debrecen, 4032 Hungary; 3 Department of Physical Geography and Geoinformatics, Faculty of Science and Technology, University of Debrecen, Egyetem sq. 1, Debrecen, 4032 Hungary; 4 MTA-DE Biodiversity and Ecosystem Services Research Group, Egyetem square 1, Debrecen, 4032 Hungary

**Keywords:** Deposition, *Celtis occidentalis*, Biomonitoring, Urban pollution, Plant sciences, Environmental sciences

## Abstract

The amount of dust deposited on tree leaves is a cost-effective indicator of air quality. Our aim was to explore the leaf surface deposition, and chlorophyll content of leaves along a road section that started at an intersection, and ended in a less disturbed suburban area in Debrecen, Hungary. We also assessed the impact of meteorological conditions on the amount of deposited dust. Leaf samples were collected in July, and September 2022 from *Celtis occidentalis*, a frequent species in green urban areas of Debrecen. We found a significant negative correlation between dust deposition, and the distance from the intersection in July. In September, dust deposition decreased considerably compared to July, due to rainfall before the second sampling. Surprisingly, we found a positive correlation between dust deposition and chlorophyll content in July. Our findings suggest that dust deposition on leaves serves as a reliable indicator of traffic intensity, because the excess dust caused by the proximity of vehicle traffic can be detected on the leaf surface. Although, rainfall can disrupt the patterns in dust deposition that have developed over an extended period through wash-off and resuspension. Hence, it is advisable to consider these effects while selecting the sampling time and evaluating the results.

## Introduction

One of the most notable air pollutants in large cities is particulate matter. Inorganic, solid particulate matter with aerodynamic diameters between 0.001 and 100 μm can occur in the air^1^. In its settled state, it is usually referred to collectively as deposited dust, which includes mineral, industrial and road dust. The main anthropogenic sources in Debrecen are traffic, domestic heating, various construction and renovation projects, and agricultural activities in the surrounding areas. Fine particles are mainly originated from combustion, while coarser particles are more likely to be the result of mechanical abrasion and resuspension^2^.

In major cities, concentrations of common pollutants are often monitored at fixed point stations^3^. The EU air quality standard for the annual PM10 concentration is 40 µg/m^3^. However, even if the current and/or average concentrations of pollutants are below the respective thresholds, the synergistic effect between them may be more harmful to living organisms than the individual pollutants. Moreover, the number of monitoring stations is often insufficient; thus, they may not be representative of the municipality as a whole^4^.

Green spaces can effectively reduce air pollutants^5^. This is one of the ecosystem services that can be directly linked to human health impacts and can even have an associated monetary value^6–8^. However, anthropogenic pollutants such as SO_2_, NO_x_, and particulate pollution can negatively affect metabolic functions, respiration, plant growth, pigmentation, and other morphological and biochemical characteristics of plants^9^. Chlorophyll is a vital pigment for photosynthetic activities and its content is usually affected by certain air pollutants, depending on the species^10^. Therefore, in sensitive species, it is a commonly used indicator of air quality.

Biomonitoring is an accessible low-cost alternative for air quality assessment, although the methods typically have the limitation that regular sample collection is needed to monitor temporal variations in air quality. Common hackberry (*Celtis occidentalis* L.) has been used in the past as a bioindicator to assess environmental pollution in urban areas^11–13^. Its wide spatial distribution makes it an appropriate candidate for biomonitoring by means of chlorophyll content and dust deposition. Dust deposition on roadside tree canopies has already been investigated, for instance, to detect barrier function^14, 15^, and to mitigate, or monitor traffic emissions^16, 17^.

Our objective was to assess how effectively a row of trees at different distances from an intersection filters particulate pollution. Intersections generally produce increased levels of pollutants due to the constant acceleration and deceleration of vehicles. The aim of our study was to analyze leaf surface deposition and chlorophyll content of roadside trees in a unique layout in Debrecen. Trees planted along a less frequented road section were sampled, where one end of this transect was connected to a busy, signalized intersection, while the other end led to a suburban area. We aimed to determine whether the excess dust generated by the vehicles could be detected on tree leaves in the presence of other urban impacts. Samples were collected once during a dry, rain-free period in July and once after rainfall in September to assess the impact of meteorological factors. Our hypotheses were: (i) the amount of dust deposition is higher on leaves collected near the intersection compared to the suburban area, (ii) after rainfall, the dust deposition is reduced compared to the dry period, (iii) chlorophyll content is higher in leaves collected in the suburban area compared to the intersection, and (iv) there is a negative correlation between dust deposition and chlorophyll content.

## Results

### Dust deposition on roadside tree leaves

**Fig. 1:**
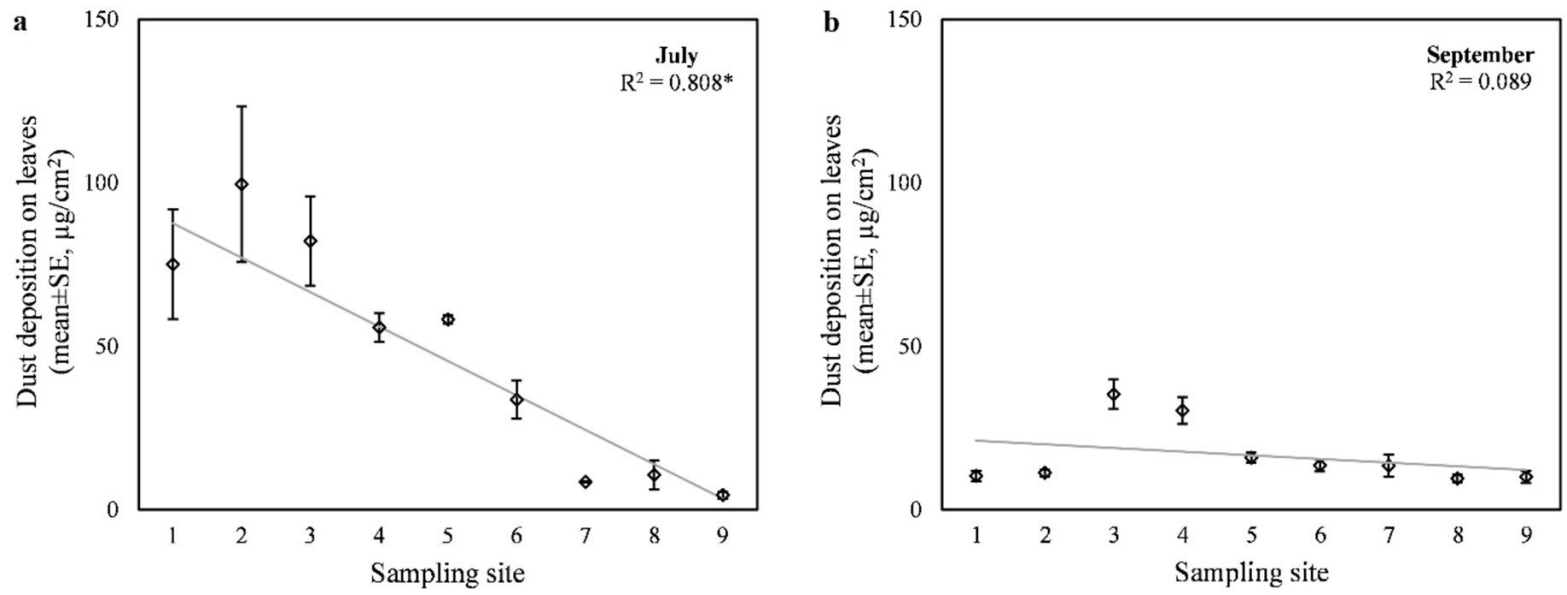
Dust deposition on leaves collected (**a**) in July and (**b**) in September, with the fitted regression line and the goodness-of-fit value (R^2^). Asterisks (*) indicate statistically significant (*p* < 0.05) relationship. The sampling sites were 50 m apart and the 1st one was closest to the intersection.

Based on the samples from July, average dust deposition ranged widely from 4.5 ± 1.7 to 99.6 ± 41.2 µg/cm^2^ along the sampled transect (Fig. 1a). There was a significant negative correlation (*r* = − 0.847, *p* < 0.001) between the dust deposition on the leaves and the distance from the intersection. We found the lowest average dust deposition at the sampling point furthest from the intersection. The trend of change was not completely monotonically linear, as e.g., the second closest point to the intersection had the highest average dust deposition. Based on the weighted regression analysis, 80.8% of the variance in dust deposition could be explained by the model (F = 105.000, *p* < 0.001). The regression model predicted an average decrease in dust deposition of 10.5 µg/cm^2^ every 50 m from the intersection, which was the approximate distance between each consecutively sampled tree.

Dust deposition on the leaf surface was significantly different between the sampling dates in July and September (t(26) = 4.557, *p* < 0.001). In September, dust deposition decreased by on average of 30.9 µg/cm^2^ compared to July (Fig. 1b). We observed the largest absolute differences at the sampling points closest to the intersection where the dust deposition was initially high in July. In this case, the correlation between dust deposition on the leaves and distance from the intersection was not significant, due to the variance and the non-linear trend of the relationship. Thus, our first hypothesis about the dust exposure near the intersection was only confirmed by the first assessment in July. Our hypothesis about the wash-off effect of precipitation was also confirmed.

### Chlorophyll content of tree leaves

**Fig. 2:**
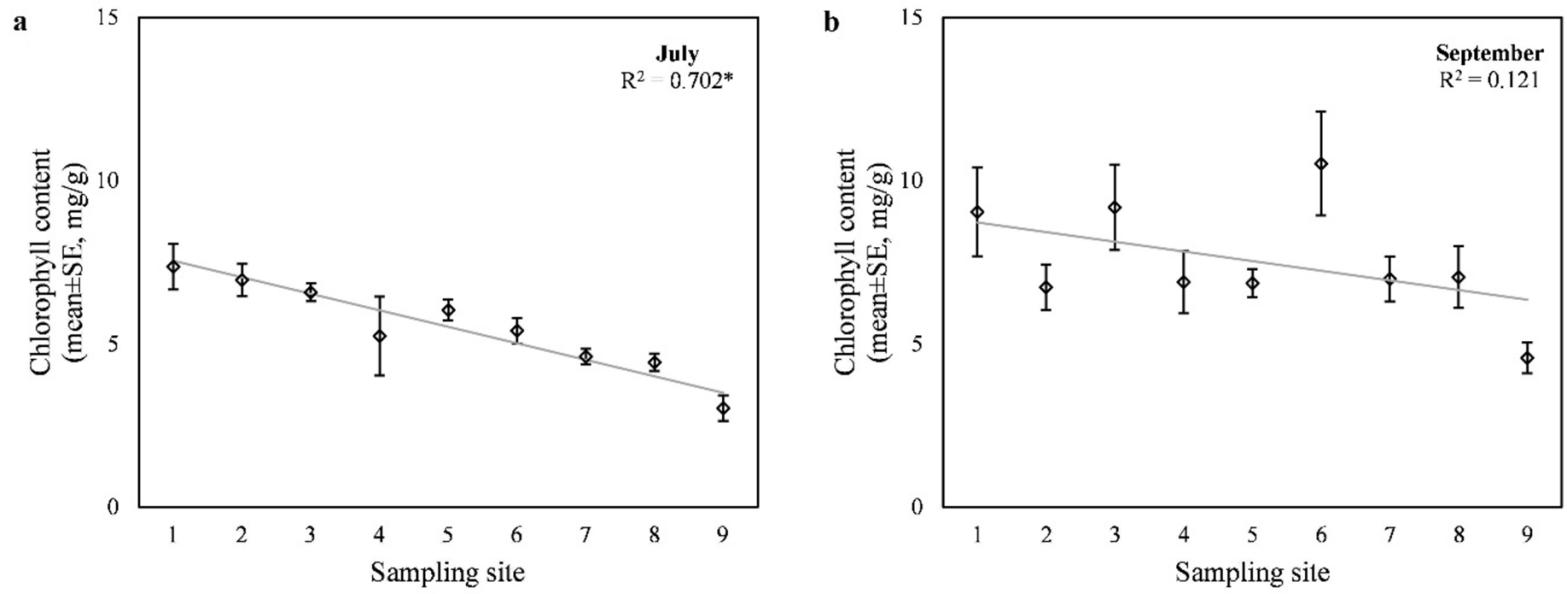
Chlorophyll content of leaves collected (**a**) in July and (**b**) in September, with the fitted regression line and the goodness-of-fit value (R^2^). Asterisks (*) indicate statistically significant (*p* < 0.05) relationship. The sampling sites were 50 m apart and the 1st one was closest to the intersection.

Similar to dust deposition, chlorophyll content also had a significant negative correlation (*r* = − 0.813, *p* < 0.001) with the distance from the traffic intersection in July. At this time of sampling, chlorophyll content ranged from 3.03 ± 0.67 to 7.37 ± 1.21 mg/g in the leaves of *C. occidentalis* (at the sampling points furthest and closest to the intersection, respectively). In this case, 70.2% of the variance in chlorophyll content could be explained by the weighted regression model (F = 58.801, *p* < 0.001) (Fig. 2a). The model predicted an average decrease in chlorophyll content of 0.51 mg/g every 50 m from the intersection. Chlorophyll content also differed significantly between the sampling dates (t(26) = − 4.385, *p* < 0.001). In September, chlorophyll content was 2.0 mg/g higher than in July on average across the sampling points (Fig. 2b). There was a considerable variability among the sampled trees, and this time the correlation with the distance from traffic was not significant. Based on these results, we rejected our hypothesis that chlorophyll content would be higher in the less disturbed area.

### The relationship between dust deposition and chlorophyll content

**Fig. 3:**
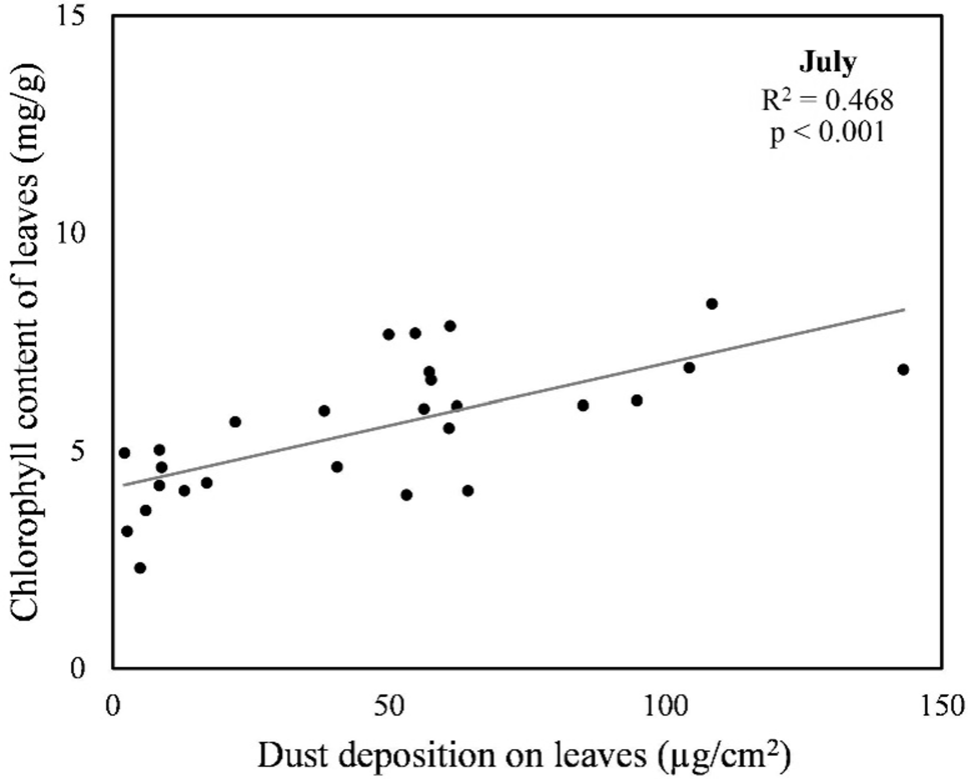
Regression plot for the dust deposition and the chlorophyll content of leaves collected in July.

Lastly, we explored the relationship between dust deposition and chlorophyll content from the same samples for the entire transect. Based on the samples collected in July, we observed a significant positive correlation (*r* = 0.684, *p* < 0.001) between the dust deposition and the chlorophyll content of the leaves, which means that as the mass of deposited dust on the leaf surface increased, the concentration of chlorophyll in the leaf tissue increased as well. Regression analysis showed a significant, but relatively weak relationship, with R^2^ = 0.468 (*p* < 0.001) (Fig. 3). In September, there was no significant correlation between dust deposition and chlorophyll content. Therefore, the last hypothesis about the negative correlation between dust deposition and chlorophyll content was rejected for both months.

## Discussion

We assessed the effects of vehicular traffic on air quality by determining the dust deposition accumulated on leaves of roadside trees. We found that the dust deposition on *C.* *occidentalis* leaves decreased with distance from the intersection, indicating continuous dilution and deposition of traffic-generated and resuspended dust with distance. The pollution source in our study was the traffic flow through the intersection near the beginning of the sampling. The road on which the samples were collected had sparse traffic; thus, its contribution to dust emissions was negligible, leaving the traffic flow through the intersection as the main source of dust emission. Differences in land cover can be used to characterize the end points of the road section. Within 100 m of first sampling point near the intersection, the main land cover types were ‘Industrial, commercial, public, military and private units’ in 42% and ‘Continuous urban fabric (sealed land: > 80%)’ in 36%. At the other end of the surveyed road section, within 100 m of sampling point 9, the main type was ‘Discontinuous dense urban fabric (sealed land: 50–80%)’ with 93%, indicating lesser anthropogenic impacts there (Fig. 5). In a meta-analysis^18^, it was concluded that leaf deposition was negatively correlated with the distance from the roadsides or any pollution source, and our result was consistent with the above statement. The concentration of solid particles naturally dilutes with distance from the road due to dispersion, which has also been studied using monitoring stations^19, 20^, but the presence of vegetation promotes this process by filtering and collecting particles. *Celtis* *occidentalis* has a high density of trichome on the leaf surface, which can result in a high dust absorbing capability^13^. Other morphological traits, such as stomata density and roughness, also contribute to the accumulation of particulate matter^21^. It is important to mention that to avoid large elevations in on-road concentrations, roadside vegetation should not be so dense that it forms a solid barrier for particles^14, 15^.

Our results in September showed an overall decrease in dust deposition compared to July. There was a rain event prior to the second sampling, which presumable led to wash-off and caused this decrease. Based on data from the Hungarian Meteorological Service, in 7 days prior to the sampling in July there was 0.3 mm of precipitation and an average daily PM10 concentration of 22.0 ± 7.0 µg/m^3^, while before the sampling in September there was 11.8 mm of precipitation and an average daily PM10 concentration of 11.5 ± 2.1 µg/m^3^^22^. The daily PM10 concentrations were significantly lower in the days prior to the sampling in September, suggesting that deposition rates may have been lower as well. However, the rate of change over time was not the same at all the sampling points. The largest decrease was observed at points closer to the intersection, where dust deposition was initially higher. There are several other environmental factors that influence the amount of dust deposition. For example, although the samples were collected from the same height, differences in canopy size and total leaf area above the sampling level had varying effects on the deposition and the transfer of dust particles during a raining event. The effect of rain is an important factor to consider when discussing dust retention by plants. The accumulation of dust particles saturates the leaf surface over time, but the amount of dust will decrease again due to rainfall and wind. Accurate measurement of this exchange is limited, and it is generally carried out using simulated rainfall in an artificial environment. For example, researchers found that particles were washed off of leaves during simulated rainfall^23^. However, another study determined that natural rainfall can also increase the accumulation of particles on the leaf surface at high concentration of PM pollution^24^. This usually occurs at low amount of rainfall, when the water-holding capacity of the leaf is not exceeded^18^. In contrast, it was even suggested that particles were not easily removed by rain once deposited on the leaf surface^25^. It can be assumed that the effect of rain wash-off will not always be obvious, and that different external circumstances can change the amount of dust deposition in distinctly different directions.

Along with the dust deposition, chlorophyll content was also determined from the same samples along the road. We found that chlorophyll content of *C. occidentalis* decreased with the distance from the traffic intersection in July; although, at a lower rate than the dust deposition did. This indicates that exposure to moderate amounts of pollutants in the air has triggered an increase in chlorophyll content. This is not necessarily an unusual finding, although several studies have reported the opposite. Air pollutants are often found to cause chlorophyll depletion of in various plant species, due to inhibition of chlorophyll synthesis or degradation of chlorophyll into pheophytin^10,26,27^. In general, urban trees are susceptible to damage, and above a certain exposure, visible alterations such as chlorosis, reduction in leaf number, leaf area, stem and root length may occur^28^. However, similarly to our results, it was found that chlorophyll in *Betula pendula* leaves was present in higher concentrations in areas with more intense anthropogenic activity^29^. It has been suggested that below a certain level of air pollutants, the chlorophyll content is positively affected due to compensatory mechanisms, e.g. to maintain the photosynthetic activity under the shading effect of particulate matter. Similar results were shown for pine and spruce species (*Pinus sylvestris* and *Picea obovata*), as moderate levels of pollution were found to stimulate chlorophyll production^30^. In another study, similar chlorophyll content was found in green wall plants at test and control sites^31^. This was explained by the innate tolerance of plants and the relatively good air quality, which also applies to the present study area. Although the underlying biochemical processes are not yet clear, these cases show that chlorophyll can increase in plants at moderate pollution levels.

Compared to July, the chlorophyll content of *C.* *occidentalis* increased significantly in September. However, the quantities determined at each location showed a high degree of irregularity in relation to each other, thus, unlike in July, no definite trend was discernible along the sampling transect in September. The chlorophyll content in the leaves of deciduous trees changes naturally with the seasons. In the local climate, it was found that chlorophyll content in the leaves of *Quercus petraea* (Matt.) Liebl. fluctuated only slightly after the initial increase at the beginning of the growing season until September^32^. However, in urban environments, anthropogenic influences modify the natural variation of chlorophyll to some extent. For example, it was found that while chlorophyll concentration in *Platanus occidentalis* L. increased slightly from June to October at an unpolluted site, it decreased at a polluted site in that same time period^33^.

The relationship between the dust deposition and the chlorophyll content can be approached from different directions. In July, the chlorophyll content has varied in parallel with the dust deposition on the leaf surface, while in September there was no clear relationship between the two parameters. This behavior usually depends on the tree species. On the one hand, tree species can show both a decrease and an increase in chlorophyll in the presence of pollutants, as described above. *Celtis* *occidentalis* is well known for its resilience in urban environments, which explains the latter behavior. On the other hand, in terms of temporal variations, an increase in chlorophyll content was observed along with a decrease in dust deposition on the leaf surface over the time of the observations. A wide range of inorganic and organic contaminants are likely to be found in the deposited dust^34^, which affect pigmentation in plants. Most of the literature on this subject comes from India where air quality is frequently poor^35^. Chlorophyll content of leaves has been reported to be lower at roadsides and industrial areas with higher levels of pollution compared to control areas^36–39^. However, dust deposition in most studies is in the magnitude of a few mg/cm^2^, while even the highest amount we found was only 99.6 ± 41.2 µg/cm^2^. Therefore, it is assumed that the air quality and dust pollution at the sampling site in Debrecen were not severe enough to cause a deficit in the chlorophyll content of the leaf tissue.

In conclusion, the dust deposition on the surface of tree leaves is a good indicator of the level of traffic-related dust pollution. However, it is very important to consider meteorological conditions in such studies. In many cases, information on precipitation is missing in the description of the sampling, which compromises the comparability of the results with others. This is also demonstrated in the present study, as the first sampling yielded the expected result for dust deposition, while the later date did not lead to a clear correlation. In terms of plant health, it can be stated that no damage was observed on *C. occidentalis* leaves as a result of the deposition of dust, which could have been indicated by the chlorophyll content. In addition, further research is needed on the relationship between chlorophyll content and particulate deposition, as different geographical factors, specific characteristics of species and varying levels of pollution in an area have a strong influence on the alteration in chlorophyll content. Therefore, any conclusion about air quality based on chlorophyll content needs careful consideration, as chlorophyll levels may increase with the dust amount deposited on the leaf surface.

Overall, it was concluded that dust deposition on the leaves is a good indicator of traffic intensity and proximity, although certain weather conditions can disrupt patterns that have developed over a longer period of time. It is therefore recommended to consider these effects when selecting sampling times and evaluating the results.

## Materials and methods

### Study area and sample collection

The study site was located in the central part of the city of Debrecen. It is at 120 m above sea level in the Great Hungarian Plain^40^. Debrecen has limited area of urban green spaces that are scattered throughout the city^41^. Currently there are no significant industrial pollution source in the region, but several major industrial investments are planned in the near future. The air quality in the city is mainly affected by the particulate pollution from vehicle traffic. Nearby agricultural activities also have a large contribution to pollution, especially from the western side, where there is no vegetation functioning as a windbreak. The city is also neighboring to the Nyírség region, which is characterized by high erodibility by wind, contributing an additional source of particulate matter^42^. The air in Debrecen exchanges slowly and is not easily renewed due to geographical and meteorological conditions and general urban effects. In addition, a large amount of mineral dust from the Sahara reaches Hungary every year^43^. Nevertheless, the annual average concentration of particulate matter, which is continuously monitored in the city, has not exceeded the health limit value in recent years.

**Fig. 4:**
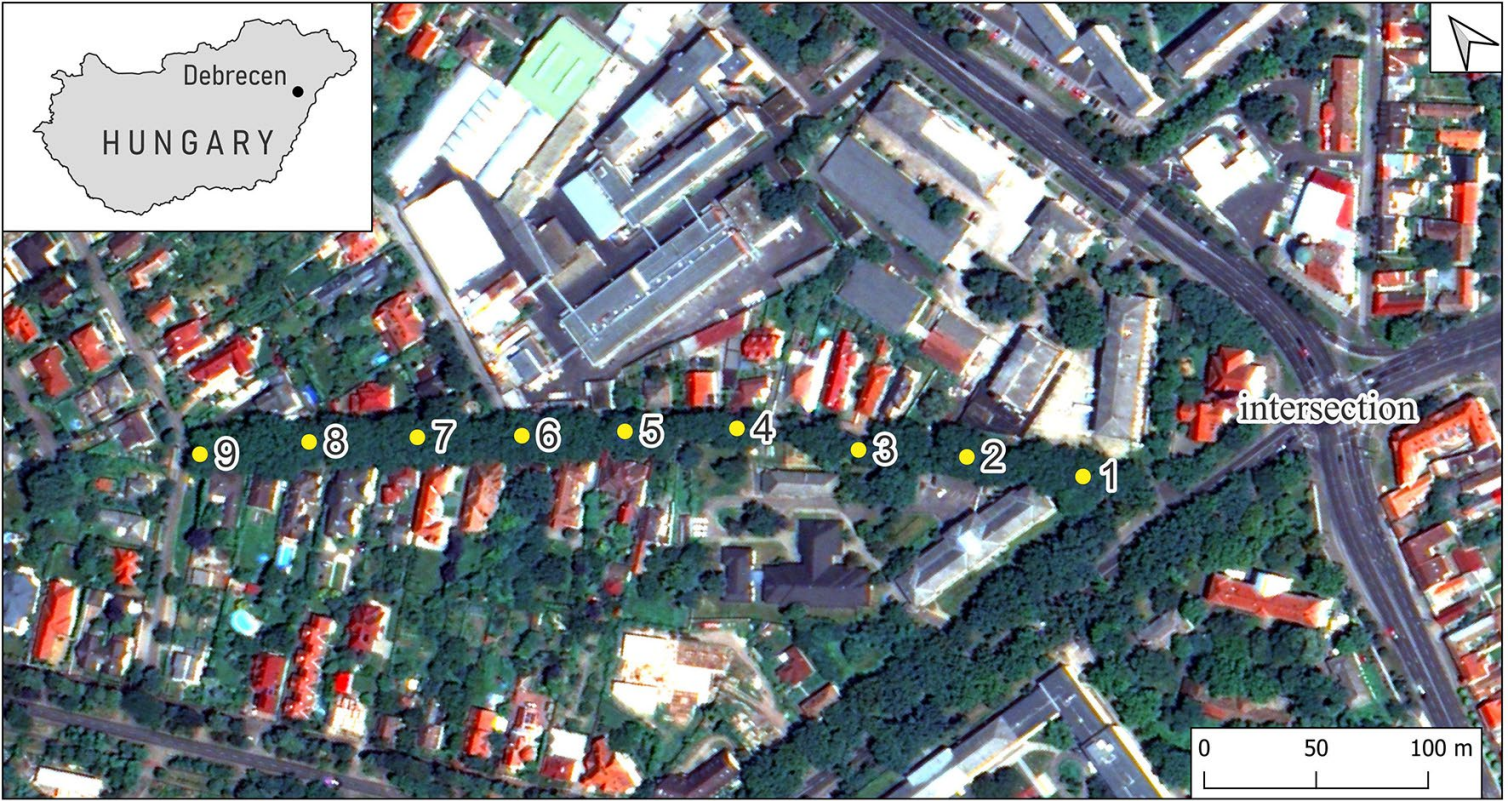
The location of sampling sites along the transect in Debrecen, Hungary. The intersection on the right is considered the most significant particulate source. The map was generated with the QGIS v.3.26 software (https://qgis.org). Background image: WorldView-2 satellite image (July 26, 2016, DigitalGlobe, Inc.).

**Fig. 5:**
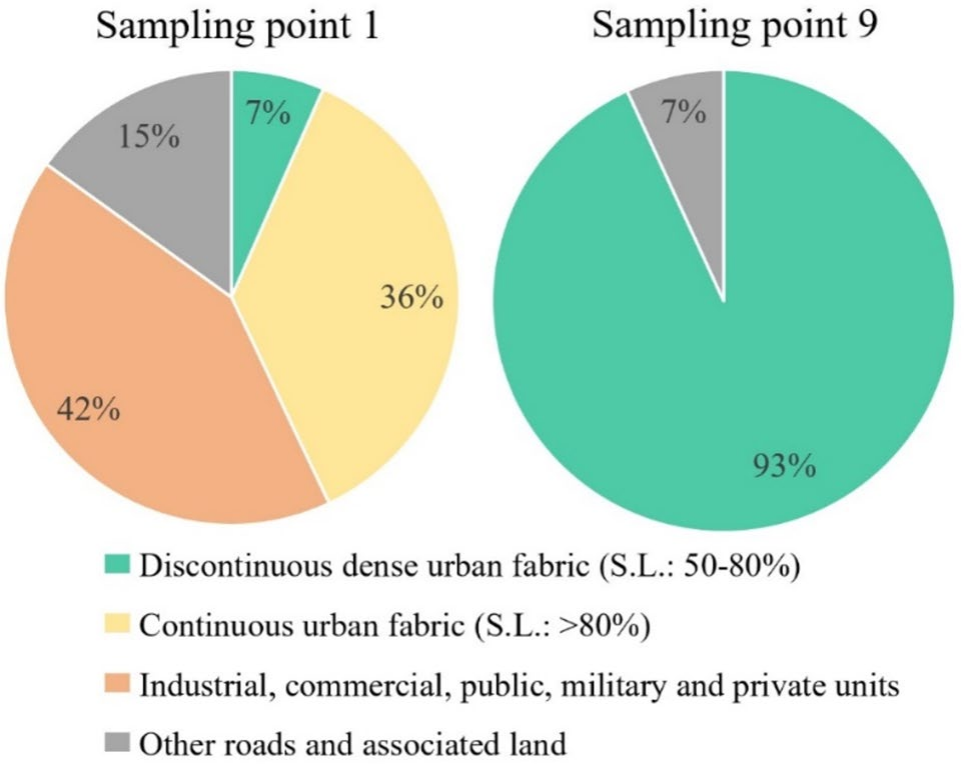
Distribution of land use types within 100 m of the first and last sampling points (S.L. = sealed land; based on data from the Copernicus Land Monitoring Service’s Urban Atlas, 2018).

**Fig. 6:**
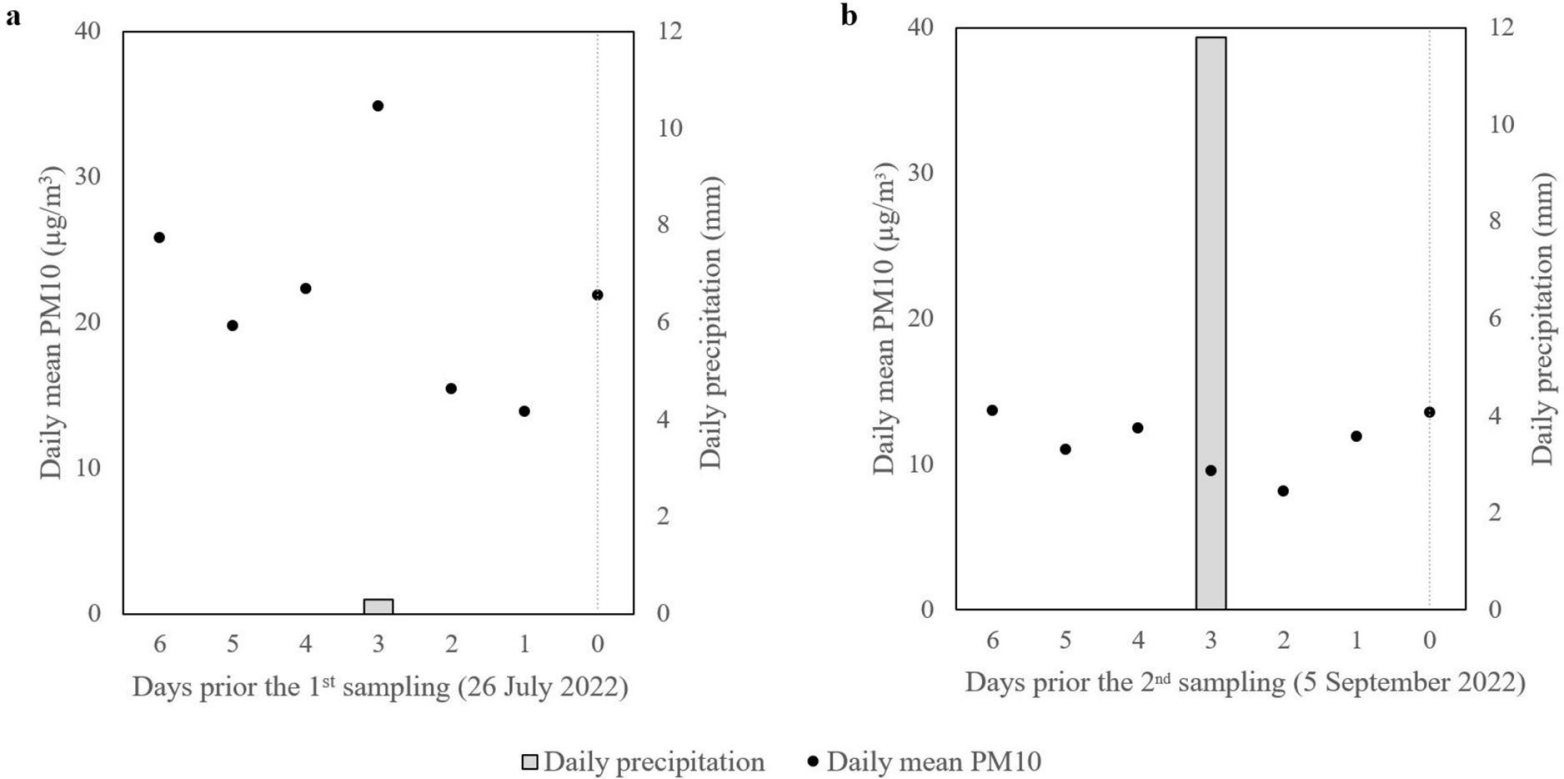
Meteorological conditions in the week prior to sample collection in (**a**) July and (**b**) September (based on data from the Hungarian Meteorological Service^22^).

Sampling was carried out along a 400 m section of a street, with a starting point approximately 100 m from a busy intersection, and an end point in a less disturbed suburban area, in July and September 2022 (Fig. 4). Land use types within 100 m of the first and last sampling points have been assessed, to characterize the difference between these end-points (Copernicus Land Monitoring Service, 2018) (Fig. 5). The Hungarian Meteorological Service reported 0.3 and 11.8 mm of precipitation and an average of 22.0 ± 7.0 and 11.5 ± 2.1 µg/m^3^ daily PM10 concentration in the weeks before the sampling in July and in September, respectively (Fig. 6)^22^. Leaf samples were taken every 50 m along the selected road section in three replicates from *C. occidentalis*, which is a deciduous tree planted along roadsides. Average-sized and healthy leaves were collected from a height of 1.5–2 m, from the side of the tree facing the road. A total of 54 samples were collected; each sample contained approximately 10 leaves. The samples were stored in paper bags and kept frozen until further processing. Species identification and sample collection were carried out by the authors. All methods including collection of plant material, were carried out in accordance with relevant guidelines and regulations. The necessary permissions and licenses needed for the collection and processing of the *C. occidentalis* were obtained.

### Analysis of dust deposition

Leaf area of each leaf was determined. Then, leaf samples were placed in 500 ml plastic containers, and mixed with 250 ml deionized water for 10 min on an analog orbital shaker (GFL 3015). The resulting suspension was filtered through a 100 μm sieve and the procedure was repeated with another 50 ml of deionized water^13, 44^. The resulting 300 ml suspension was then filtered through a vacuum pump (BOECO R-300) using filter paper with a retention diameter of 5–8 μm (Munktell 392, Ahlstrom), previously weighed on an analytical balance (ME, METTLER TOLEDO). The filter papers were reweighed with the collected dust to obtain the net dust mass, which was converted to µg/cm^2^ of leaf area.

### Analysis of chlorophyll content

For the analysis of chlorophyll content, approximately 20 mg of fresh leaf tissue was prepared from each sample. The leaf tissue was crushed in a mortar and homogenized in 5 ml of 96% (v/v) ethanol. The extracts were centrifuged at 1500 rpm for 3 min (IEC Centra MP4). Absorbance was measured using spectrophotometric analysis (BOECO S-220) at wavelengths of 653, 666, and 750 nm against 96% (v/v) ethanol blank. Chlorophyll content was calculated based on *Eq. 1*, where *V* is the extract volume (l), *m* is the fresh weight of leaf tissue (g), and *E666* and *E653* are the absorbances at 666 nm and 653 nm minus the absorbance at 750 nm, respectively^45^.

1 $${\text{Chlorophyll }}\left( {{\text{mg}}/{\text{g}}} \right){\text{ }} = {\text{ }}\left( {{\text{2}}.{\text{57 }} \times {\text{ E666 }} + {\text{ 23}}.{\text{6 }} \times {\text{ E653}}} \right){\text{ }} \times {\text{ V }}/{\text{ m}}$$

### Statistical analysis

We used SPSS Statistics 21 software (IBM Corp., Armonk, NY, USA^46^) for statistical analyses. Normal distribution of the variables was checked by the Shapiro–Wilk test. Pearson’s correlation coefficient was used to reveal the relationship among variables (distance from sampling site, dust deposition and chlorophyll content). Simple regression analysis was used to describe the relations between the variables. When error variances showed heteroscedasticity weighted least squares regression was used, where the weights were based on the reciprocal of the variance of the given variable. The difference between samples in July and September was tested using paired t-tests.

## Data Availability

The datasets generated during and/or analyzed during the current study are available from the corresponding author on reasonable request.
